# Conserved CO-FT regulons contribute to the photoperiod flowering control in soybean

**DOI:** 10.1186/1471-2229-14-9

**Published:** 2014-01-07

**Authors:** Chengming Fan, Ruibo Hu, Xiaomei Zhang, Xu Wang, Wenjing Zhang, Qingzhe Zhang, Jinhua Ma, Yong-Fu Fu

**Affiliations:** 1 MOA Key Lab of Soybean Biology (Beijing), National Key Facility of Crop Gene Resource and Genetic Improvement, Institute of Crop Sciences, Chinese Academy of Agricultural Sciences, 12 Zhongguancun Nandajie, Haidian District, Beijing 100081, China; 2 Institute of Genetics and Developmental Biology, Chinese Academy of Sciences, Beijing, China; 3 CAS Key Lab of Biofuels, Shandong Provincial Key Lab of Energy Genetics, Qingdao Institute of BioEnergy and BioProcess Technology, Chinese Academy of Sciences, Qingdao, Shandong 266101, China

**Keywords:** *CONSTANS*, *FLOWERING LOCUS T*, Paralog, Ortholog, Functional divergence, Soybean

## Abstract

**Background:**

*CO* and *FT* orthologs, belonging to the BBX and PEBP family, respectively, have important and conserved roles in the photoperiod regulation of flowering time in plants. Soybean genome experienced at least three rounds of whole genome duplications (WGDs), which resulted in multiple copies of about 75% of genes. Subsequent subfunctionalization is the main fate for paralogous gene pairs during the evolutionary process.

**Results:**

The phylogenic relationships revealed that *CO* orthologs were widespread in the plant kingdom while *FT* orthologs were present only in angiosperms. Twenty-eight *CO* homologous genes and twenty-four *FT* homologous genes were gained in the soybean genome. Based on the collinear relationship, the soybean ancestral *CO* ortholog experienced three WGD events, but only two paralogous gene pairs (*GmCOL1*/*2* and *GmCOL5*/*13*) survived in the modern soybean. The paralogous gene pairs, *GmCOL1*/*2* or *GmCOL5*/*13,* showed similar expression patterns in pair but different between pairs, indicating that they functionally diverged. *GmFTL1* to 7 were derived from the same ancestor prior to the whole genome triplication (WGT) event, and after the Legume WGD event the ancestor diverged into two branches, *GmFTL3*/*5*/*7* and *GmFTL1*/*2*/*4*/*6. GmFTL7* were truncated in the N-terminus compared to other *FT*-lineage genes, but ubiquitously expressed. Expressions of *GmFTL1* to *6* were higher in leaves at the flowering stage than that at the seedling stage. *GmFTL3* was expressed at the highest level in all tissues except roots at the seedling stage, and its circadian pattern was different from the other five ones. The transcript of *GmFTL6* was highly accumulated in seedling roots. The circadian rhythms of *GmCOL5*/*13* and *GmFT1*/*2*/*4*/*5*/*6* were synchronized in a day, demonstrating the complicate relationship of *CO*-*FT* regulons in soybean leaves. Over-expression of *GmCOL2* did not rescue the flowering phenotype of the *Arabidopsis co* mutant. However, ectopic expression of *GmCOL5* did rescue the *co* mutant phenotype. All *GmFTL1* to *6* showed flower-promoting activities in *Arabidopsis*.

**Conclusions:**

After three recent rounds of whole genome duplications in the soybean, the paralogous genes of *CO-FT* regulons showed subfunctionalization through expression divergence. Then, only *GmCOL5/13* kept flowering-promoting activities, while *GmFTL1* to 6 contributed to flowering control. Additionally, *GmCOL5*/*13* and *GmFT1*/*2*/3/*4*/*5*/*6* showed similar circadian expression profiles. Therefore, our results suggested that *GmCOL5*/*13* and *GmFT1*/*2/3*/*4*/*5*/*6* formed the complicate *CO-FT* regulons in the photoperiod regulation of flowering time in soybean.

## Background

The photoperiod pathway, which includes a number of genes that form its core, as well as input and output genes, is very important for angiosperms to flower at a precise time in a year [1]. The circadian-regulated gene *CONSTANS* (*CO*) is a central regulator of this pathway, which coordinates light and clock inputs in leaves to trigger the expression of florigen gene *FLOWERING LOCUS T* (*FT*) [2,3]. In *Arabidopsis*, a long-day (SD) plant, the transcript peak of *CO* mRNA occurs late in the day in LD, but after dusk in SD [4]. CO protein, in turn, is stabilized by light and rapidly degrades in darkness, and activates the expression of *FT* in LD conditions [5,6]. In rice, a short-day (SD) plant, *Hd1,* the *CO* ortholog, functions in the promotion of *Hd3a* (the *FT* ortholog) expression in SD conditions, but in the inhibition of *Hd3a* expression in LD conditions [7,8]. *Hd1* mRNA begins to accumulate after dusk and decrease before dawn [8]. In *Populus trichocarpa*, *CO*-*FT* regulon also plays a pivotal role in flowering and controlling of a highly adaptive trait for forest trees [9]. The day-length flowering response in temperate cereals, such as wheat and barley, appears to involve the activation of an *FT* and *FT-like 1* (*FT1*) [10]. *TaHD1* (a *CO-like* gene in wheat) can complement the rice *hd1* mutant [11], and *LpCO3* (a *CO-like* gene in *Lolium perenne*) can rescue the *co* mutant phenotype [12]. Thus, the *CO-FT* regulon is conserved among angiosperms analyzed, even though it has different modes in different species.

*CO* homologs belong to B-box family (BBX) family and are conserved in plants including algae [13-16]. The BBX (Pfam: PF01161) represents a subgroup of zinc finger proteins, which contain one or two B-box domains mediating protein-protein interactions in animals, yeast, and plants [17,18]. Besides B-box domains in the N-termini, some members of BBX family have a C-terminal CCT domain, which includes a nuclear import signal [4] and a domain of interaction with the ubiquitin ligase COP1 [6]. *CO* homologs can be sub-grouped into three major sub-types: type I with two B-box domains, type II with one B-box domain, and type III with one B-box domain and one degraded B-box domain [2,15,16]. Some members of type I genes, such as *CO* in *A. thaliana*, *Hd1* in rice, and *PnCO* in *Pharbitis nil*, control flowering in different plants [12,14,15,19-25]. The *CO* homolog is also found in algae. *CrCO* from *Chlamydomonas reinhardtii* can complement the *Arabidopsis co* mutant and promote flowering [16], indicating the function of *CO* orthologs is ancient and conserved.

Phosphatidyl ethanolamine-binding protein family (PEBP, Pfam: PF00643) has now been identified in many kingdoms and their basic structures as well as sequences are evolutionarily conserved [26]. In plants, PEBP genes are mainly classified into three clades: *FT-like*, *TFL-like* and *MFT-like* clades [27]. *MFT-like* is ancestral to the other two clades and shown to be involved in the development of reproductive tissues in moss or seed development and germination in seed plants [28-31]. Several members of the *TFL-like* clade, such as *CEN* from *Antirrhinum*[32] and *TFL1* from *Arabidopsis*, have important roles in delaying flowering and maintaining indeterminacy of inflorescence meristem [33]*.* As a major component of florigen, *FT-like* genes mediate the onset of flowering through the photoperiod pathway, vernalization pathways, and other pathways in all angiosperms examined [34-36]. *FT/TFL1-like* genes, such as *PaFTL1* and *PaFTL2*, code for proteins with a *TFL1-like* function in gymnosperms [30]. Taken together, the first duplication event resulting in two families of plant PEBP genes (*MFT-like* and *FT/TFL1-like*) seems to coincide with the evolution of seed plants, in which independent control of bud and seed dormancy is required [30]. The second duplication resulting in the production of the *FT-like* and *TFL1-like* clades probably coincides with the evolution of angiosperms [30]. In addition, the similarity of amino acid among the *FT-* and *FTL-like* clades is high, and key amino acids are responsible for this functional divergence [37-39].

Gene duplications have occurred during plant speciation, and the generation of several paralogous copies allows gene diversification. Paralogs may retain the function of the ancestral genes, and thus act redundantly and/or additively due to the increased protein dosage. But they may also develop non-, sub- or neo-functions [40]. In soybean, about 75% genes are present in multiple copies [41], and about 50% of paralogs are differentially expressed. Most of them have undergone sub-functionalization and only a small proportion of the duplicated genes have been neo-functionalized or non-functionalized [42,43].

In this study, the evolutionary relationship between the BBX or PEBP gene family and plant speciation was investigated at the genome level. And then *CO* and *FT* orthologs were screened in the soybean genome. Based on the phylogenetic and the collinear relationship, 4 of *CO* orthologs (*GmCOL1*, *2*, *5*, and *13*) and 6 of *FT* orthologs (*GmFTL1* to *6*) were identified in the soybean. Finally, the detailed expression profiles of these genes in soybean and their flowering functions in *Arabidopsis* were analyzed. The results suggest that in soybean there were more than one *CO* and *FT* orthologs with the function of flowering control.

## Results and discussion

### *CO-like* genes are ancient, whereas *FT-like* genes are recent in plants

**Figure 1:**
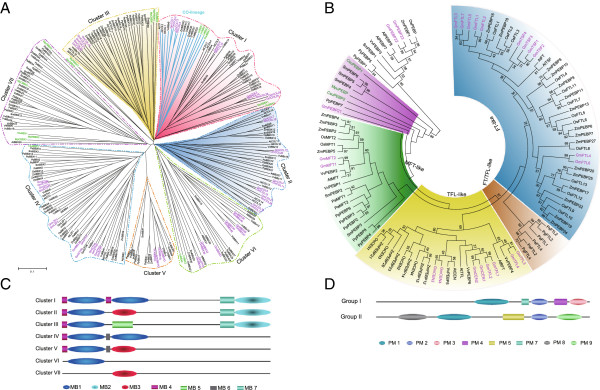
**Phylogentic trees of plant BBX or PEBP family.** The phylogenetic tree of plant BBX or PEBP genes reconstructed by MEGA 5.0 with the NJ method and the bootstrap test (1000). Soybean BBX or PEBP genes from soybean were in purple and from the algae in green. **A**, the phylogenetic tree of plant BBX gene family. The *CO* homologs were in different color background, and the blue branches were the candidate *CO*-lineage genes. **B**, the phylogenetic tree of plant PEBP gene family. Group I was in color background and Group II had no color. The motif organizations of the BBX or PEBP family based on the results of MEME and MAST, and different motifs were in different color, and the best match sequences was listed in Additional file 2. **C**, the motif organizations of different clusters of the BBX family. **D**, The motif organizations of different groups of the PEBP family.

**Figure 2:**
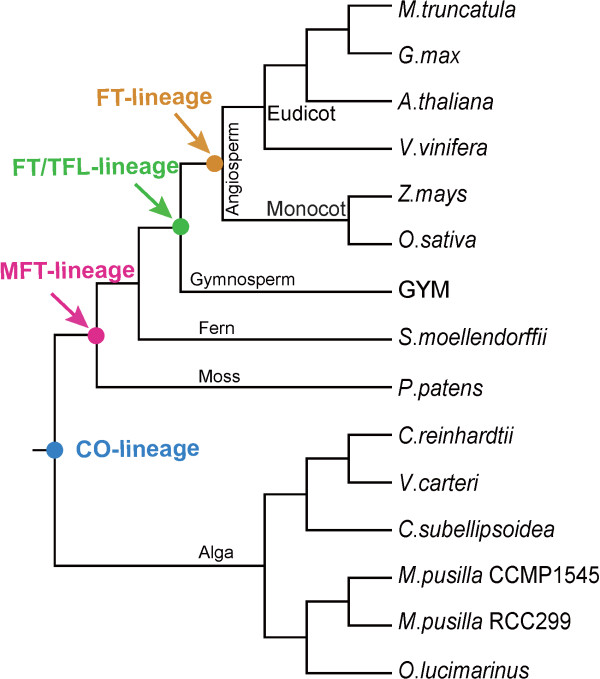
**The evolutionary processes of *CO*-lineage or *FT*-lineage.** The blue, red, green and orange solid circle showed the earliest possible occurrence of *CO*-, *MFT*-, *FT/TFL*-, and *FT*-lineage during the evolutionary process of the plant, respectively. GYM was the gymnosperm, such as *Picea sitchensis*, *Pinus radiate*, *P. pinaster* and *P. sylvestris*.

The profile-HMMs for the BBX family (PF00643) and the PEBP family (PF01161), including *CO-like* and *FT-like* genes, respectively, were employed through HMMER to search candidate genes of the two families in plants with available genomes, including two monocots (*Oryza sativa* and *Z*ea *mays*), three eudicots (*Vitis vinifera*, *Arabidopsis thaliana*, and *Glycine max*), four gymnosperms (*Picea sitchensis*, *Pinus radiate*, *Pinus pinaster*, and *Pinus sylvestris*), one lycophyte (*Selaginella moellendorffii*), one moss (*Physcomitrella patens*), and six chlorophytes (*Ostreococcus lucimarinus*, *Micromonas pusilla* RCC299, *M. pusilla* CCMP1545, *Coccomyxa subellipsoidea*, *Volvox carteri*, and *Chlamydomonas reinhardtii*) (Additional file 1). Phylogenetic trees of the *CO-like* and *FT-like* gene families were similarly reconstructed by MEGA 5.0 with Neighbor-joining method (Figure 1A and B). MEME and MAST (http://meme.nbcr.net) were employed to investigate motifs and their organizations among different clusters of the BBX or PEBP family, respectively (Figure 1C and 2D).

Different BBX clusters had completely diverged before the divergence of bryophytes and pteridophytes [13]. According to the phylogenetic tree (Figure 1A) and their own motif organizations (Figure 1C, Additional file 2), the plant BBX family was grouped into seven clusters, Cluster I through VII. Among them, Cluster I, III, IV, VI, and VII can be found in the unicellular green algae and Cluster II and V first appeared in the moss plant. Thus, seven BBX clusters appeared prior to the occurrence of land plants. Based on the alignment results of SMART (http://smart.embl-heidelberg.de/), motifs MB1 and MB4 were equivalent to the B-box1 domain, and MB3 or MB6 to the B-box2 domain (a degraded B-box [15,16]), and MB2 and MB7 to the CCT domain. *CO* homologs contained conserved B-box1 domain and CCT-domain [4,15].The members of Cluster I, II and III also had both B-box1 and CCT-domain, suggesting that the members of the three clusters were the *CO* homologs. In addition, BBX Cluster I contained *CO* in *Arabidopsis*[14], *Hd1* (*OsBBX12*) in rice [20], and *CrCO* (*CrBBX1*) in a green algae [16]. Thus, BBX Cluster I contained the *CO* orthologs from different species (Figure 1A), indicating that they formed the conserved motifs and functioned prior to the divergence of algae and plants and were monophyletic.

*CO* homologs probably represented as ancient regulators of photoperiod-dependent events [16]. Functionally, *CrCO* from *C. reinhardtii* shows important roles in processes regulated by the photoperiod and the circadian clock [16]. In the moss *P. patens*, *PpCOL1* (PpBBX6 in this study) expression is controlled by the circadian clock [44]. Transcripts of *PaCOL1* and *PaCOL2* in *Picea abies* can also be regulated by the photoperiod [45]. For the flowering plants, *CO* and *Hd1*, display conserved functions in regulating the flowering time through affecting transcriptions of *FT* or *Hd3a* under the LD or SD conditions, respectively [14,20].

For the plant PEBP gene family, the members could be grouped into two groups, Group I and II, with conserved motif organization, respectively (Figure 1B and D, Additional file 2). *MFT-like*s, *FT/TFL-like*s, *TFL-like*s*,* and *FT-like*s belonged to Group I. Based on the phylogenetic tree (Figure 1B), *MFT-like* genes were presented in all the land plants, and may be the ancestral form of *FT/TFL-like*, *TFL-like*, and *FT-like* genes [28]. *P. patens* had only *MFT-like* genes, whose expressions were regulated by circadian rhythm with maximum expressions in gametangia and sporophytes, indicating an involvement in the development of reproductive tissues in the moss [28]. Similarly, the *MFT-like* genes display important roles in the seed development or dormancy in angiosperms, but do not affect the flowering time [29,31,46]. Before the appearance of seed plants, the function divergence of *FT-like* genes and *TFL-like* genes is not obvious, and the function of some PEBP genes is close to *TFL-like* genes, although their sequences and key motifs are much similar to that of *FT-like* genes [30,45]. Only in angiosperms, the function divergence of *FT-like* genes (as an activator) and *TFL-like* genes (as a repressor) is significant as oppositely regulating the flowering time in monocots and eudicots [38,47-54]. In addition, *TSF* not only plays a role as a floral promoter in the photoperiod pathway redundantly with *FT*, but also makes a distinct contribution to *Arabidopsis* flowering in SD conditions. *TSF* overexpression causes a precocious flowering phenotype independent of photoperiods and *CO* or *FLC*, indicating *FT* and *TSF* are differently regulated by distinct floral-inducing signals [55,56]. All above, *FT-like* genes are present as the main flowering regulator after the divergence of angiosperms and gymnosperms and show different functions from that of *TSF-like* genes.

Taken together, *CO*-lineage genes were present in different plants from the unicellular green alga to the flowering plant (Figure 2), and their functions were ancient and conserved. However, *FT*-lineage genes were functionally diverged from *MFT*-like or *TFL*-like genes (Figure 2) when flowering plants occurred. Thus, *FT* orthologs appeared later than *CO* orthologs, and the *CO-FT* regulon was a product of a very long evolutionary process. But the mechanism of *CO* regulating the *FT* transcription was conserved in the angiosperm, and they functioned together as a *CO*-*FT* regulon in regulation of the flowering time through a photoperiod-dependent mode [2,57].

### Duplications of the *CO*-*FT* regulon in the soybean evolution

**Figure 3:**
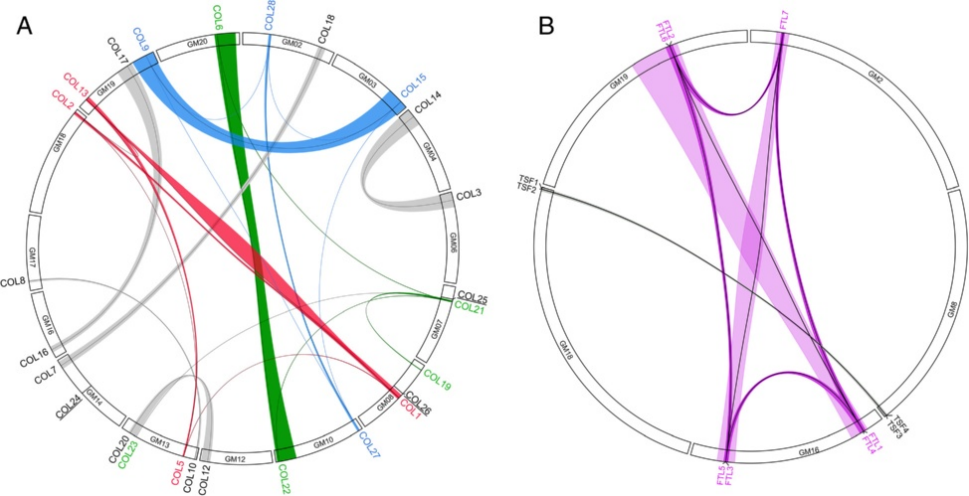
**The collinear relationships of homologous blocks containing *CO-like *or *FT-like *genes. A**, the collinear relationships among soybean *CO-like* genes *GmCOL1*/2/*5*/*13*, *GmCOL6*/*19*/*21*/*22*/*23*, and *GmCOL9*/*15*/*27*/*28* were in red, green, and blue, respectively. The gray rainbows showed the collinear relationship arose only after the *Glycine* WGD event. Singleton genes were underlined. **B**, The collinear relationships among soybean *FT-* lineage genes. The rainbow in purple showed the collinear relationship among soybean *FT*-lineage genes, and that in green displayed the collinear relationships among soybean *TSF*-lineage genes.

In the soybean genome, 28 *CO-like* genes in total, named as *GmCOL1* through *28*, can be grouped into Cluster I, II and III (Figure 1A), and most of them except for *COL24*, *25*, and *26* experienced WGD (Figure 3A). Three loci experienced the Gamma WGT and two WGD events, and then resulted in *GmCOL1*/2/*5*/*13* (Cluster I), *GmCOL6*/*19*/*21*/*22*/*23* (Cluster II), and *GmCOL9*/*15*/*27*/*28* (Cluster III), respectively (Figure 3A). Others were divergent after the *Glycine* WGD event. Evolutionarily, the soybean *CO* orthologs may be anyone of Cluster I, II, and III. However, based on the phylogenetic tree, *GmCOL1*, *2*, *5*, and *13* among 28 *CO-like* genes were much closer to *CrCO*, *CO*, and *Hd1* (Figure 1A), which showed flowering activity in plants [16,20]. Additionally, the syntenic blocks containing *GmCOL1* or *2* and *GmCOL5* or *13* in chromosomes were divergent after the legume WGD event according to the average *Ks* values of homologous blocks (0.3 ≤ *Ks* ≤ 1.5) (Table 1). Therefore, *GmCOL1*, *2*, *5,* and *13* were the good candidates of *CO* orthologs in the soybean, which was consistent with Jung *et al*. [58]. Therefore, they were selected for further study here.

**Table 1:** **Collinear relationships of homologous blocks containing *CO *** **or *FT *** **orthologs in soybean**

**Gene 1**	**Block 1**			**Gene 2**	**Block 2**			**Averange *Ks***
	**Chr.**	**Start1 (bp)**	**Stop1 (bp)**		**Chr.**	**Start2 (bp)**	**Stop2 (bp)**	
*GmCOL1*	GM08	20,564,643	23,533,251	*GmCOL2*	GM18	59,120,324	60,758,130	0.1804
*GmCOL1*	GM08	22,488,108	22,903,468	*GmCOL5*	GM13	7,094,033	7,353,077	0.7170
*GmCOL1*	GM08	22,268,496	22,705,283	*GmCOL13*	GM19	5,037,623	5,784,423	0.7074
*GmCOL2*	GM18	59,759,300	60,359,904	*GmCOL5*	GM13	6,762,198	7,353,077	0.6861
*GmCOL2*	GM18	59,768,883	60,286,871	*GmCOL13*	GM19	4,269,582	5,784,423	0.6852
*GmCOL5*	GM13	4,686,036	7,380,393	*GmCOL13*	GM19	1,047,196	6,070,543	0.2339
*GmFTL7*	GM02	3,884,100	7,368,892	*GmFTL3, 5*	GM16	26,025,808	32,876,516	0.2402
*GmFTL7*	GM02	5,819,578	6,251,781	*GmFTL2, 6*	GM19	35,107,064	36,353,817	1.020
*GmFTL7*	GM02	6,298,304	5,732,540	*GmFTL1, 4*	GM16	3,729,118	5,022,006	0.8591
*GmFTL3, 5*	GM16	30,464,871	31,024,893	*GmFTL2, 6*	GM19	35,107,064	36,353,817	0.9123
*GmFTL3, 5*	GM16	30,321,165	31,089,979	*GmFTL1, 4*	GM16	5,016,992	9,730,561	0.7530
*GmFTL2, 6*	GM19	27,993,447	37,257,559	*GmFTL1, 4*	GM16	6,662,519	3,315,381	0.2702

Note: The homologous blocks, containing *CO* or *FT* orthologs, were gained by MCScanX. Average *Ks* values of homologous blocks were the mean of *Ks* values of paralogous gene pairs in blocks.

There were 11 *FT-like* genes in the soybean (Figure 1B), and according to the collinear relationships (Figure 3B) they can be grouped into two clades, one including *GmFTL1* to *7* and the other composing of *GmTSF1* to *4*. Compared with the previous results of Kong *et al.*[59], *GmFTL1* to *6* were equivalent to *GmFTL3a*, *3b*, *2a*, *5a*, *2b* and *5b*, and *GmTSF1* to *4* corresponded to *GmFTL1b*, *1a*, *6* and *4*, respectively. *GmFTL1*-*6* all experienced WGDs as well as tandom duplications (Figure 3B). *GmFTL7* with only a shortened PEBP domain and lacking the N-terminal segment was diverged from its paralogous gene *GmFTL3* (Table 1). However, *GmFTL7* was strongly expressed in most tissues detected and induced by the photoperiod (Additional file 3). *GmFTL3* (*GmFTL2a*) and *GmFTL4* (*GmFTL5a*) coordinately control flowering and enable the adaptation of soybean to photoperiodic environments [59,60]. In *Arabidopsis*, *FT* mainly functions in LD while *TSF* makes a distinct contribution only in SD conditions [56,61,62], indicating the function of *FT* and *TSF* is divergent in regulating the flowering time. In soybean, *GmTSF1* and -2 displayed much similar sequences with *TSF. GmTSF3*/*4* showed much similar sequences with *FT* than that with *TSF* (Additional file 4), but they should be the *TSF* lineage according to the collinear relationship (Figure 3B). Furthermore, ectopic expression of *GmTSF3* and *GmTSF4* in *Arabidopsis* did not have flower-promoting activities under LD conditions (Additional file 5), so did *TSF* in *Arabidopsis*. Thus, *GmFTL1* to *6* were here selected as soybean *FT* orthologs for further study.

### Expression divergences among the soybean *CO* and *FT* paralogs showing spatio-temporal functions of *CO-FT* regulons

**Figure 4:**
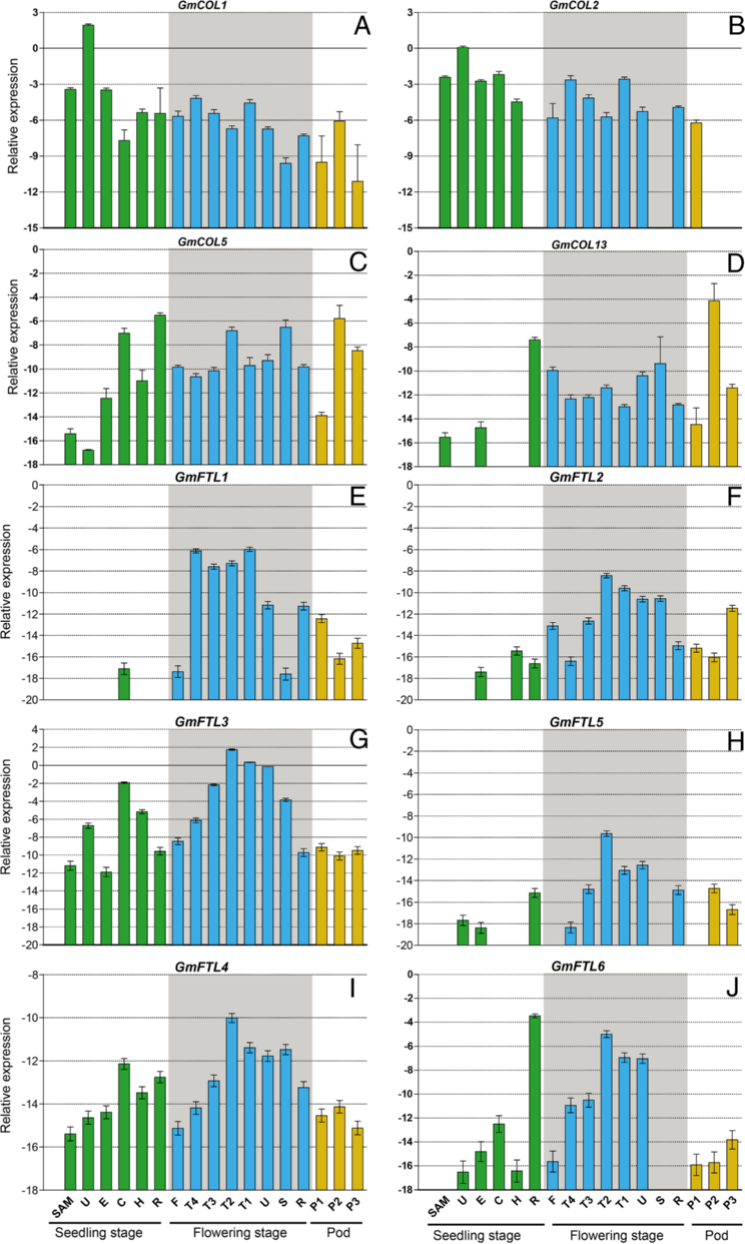
**Expression profiles of soybean *CO *or *FT *orthologs. A**, **B**, **C** and **D** was expression profile of *GmCOL1*, *2*, *5* and *13*, respectively. **E**, **F**, **G**, **H**, **I** and **J** was expression profile of *GmFTL1*, *2*, *3*, *5*, *4* and *6*, respectively. R, root; H, hypocotyl; C, cotyledon; E, epicotyl; U, unifoliolate leaf; S, stem; T1, T2, T3, T4, the first, second, third and fourth trifoliolate leaf, respectively; F, flower; SAM, the shoot apex (including the apical meristem and immature leaves) at the seedling stage. P1, P2, and P3: seven, fourteen and twenty one days after the onset of flowering, respectively. The geometric means of *GmACT11* and *GmUKNI* transcripts were used as the reference transcript.

In soybean, spatio-temporal expression profiles of four candidate *CO* orthologs (Figure 4A-D) and six *FT* orthologs (Figure 4E-J) were investigated by quantitative real time RT-PCR at the stages of seedling and flowering under SD conditions (8 h light/16 h dark).

The transcript of *GmCOL1*/*2* accumulated much more than that of *GmCOL5*/*13* in most tissues tested, and the expressions of *GmCOL1* and *GmCOL5* did not show tissue-specific, while *GmCOL2* and *GmCOL13* displayed distinct spatio-temporal expression patterns. For example, the expression of *GmCOL2* was not detected in roots at the seedling stage, in the stems at flowering, and in pods at 14 and 21 DAF (Days After Flowering) (Figure 4B). And transcripts of *GmCOL13* were undetectable in unifoliolates, cotyledons, and hypocotyls (Figure 4D). *GmCOL1*, 2*,* and *5* were expressed in cotyledons and unifoliolates at the seedling and flowering stages (Figure 4A, B and C). For the photoperiod-sensitive plant, the photoperiodic signals at the seedling stage are important to regulate flowering time. These results indicated that *GmCOL13* may not be the key gene of photoperiodic responses during the early stage of floral induction in soybean.

The expressions of *GmFTL1* and *GmFTL2* were undetectable in unifoliolates, but they strongly expressed in trifoliolates at the flowering time, and transcripts of other four *GmFTL*s were lower in leaves at the seedling stage than that at the flowering time (Figure 4E-J). So, soybean *GmFTL* genes were induced along with developmental progress. Amongst the six soybean *FTL* genes, *GmFTL3* showed higher expression level compared to that of the other genes in most of the tissues examined (Figure 4G), suggesting that *GmFTL3* was very important to promote flowering in soybean, as indicated by Kong *et al*. [59]. *GmFTL4* also was constitutively expressed but at relatively lower level compared with *GmFTL3*. In the seedling stage, *GmFTL3* and *4* were expressed at higher levels than their paralogs, *GmFTL5* and *GmFTL6*, respectively. *GmFTL1*, *3*, and *4* were strongly expressed in cotyledons (Figure 4E, G and I), which can produce sufficient FT proteins to induce flowering in *Arabidopsis*[63], suggesting that these three genes were important for floral induction at the early stages of soybean development. *GmFTL5* was expressed at low levels and was not detected in shoot apical meristems (SAM), cotyledons, and hypocotyls at the seedling stage as well as stems at the flowering stage (Figure 4H). The expression of *GmFTL6* was the highest one among six soybean *FTL* genes in roots at the seedling stage, but no expressions were detected in roots at the flowering stage (Figure 4J). Noticeably, expressions of *GmFTL1*, *2*, *3*, *4*, *6* were detected in flowers, and *GmFTL1*, *2*, *3*, *4*, *5* and *6* were expressed in pods (Figure 4), suggesting that *FTL* genes kept the ancient function of the PEBP family and may be important in reproductive development.

*CO* regulates *FT* mainly in leaves, the receptors of photoperiod signals. Soybean unifoliolates only were competent for receiving the signal of SD to promote flower initiation and 3 days of short-day treatment were sufficient for floral induction [64]. As Figure 4 shown, the transcripts of *GmCOL1*/*2*/*5* and *GmFTL3*/*4*/*5*/*6* were detected in unifoliolate leaves at the seedling stage. In addition, cotyledons were shown to be another receptor of photoperiod signals besides leaves [63]. Expectedly, *GmCOL1*/*2*/*5* and *GmFTL1*/*2*/*3*/*4*/*6* were expressed in the cotyledons at the seedling stage (Figure 4). Combined results indicated that *GmCOL1*/*2*/*5* and *GmFTL3*/*4*/6 had important roles in response to photoperiod at the soybean seedling stage.

### The circadian rhythm of soybean *CO*-*FT* regulons in leaves

**Figure 5:**
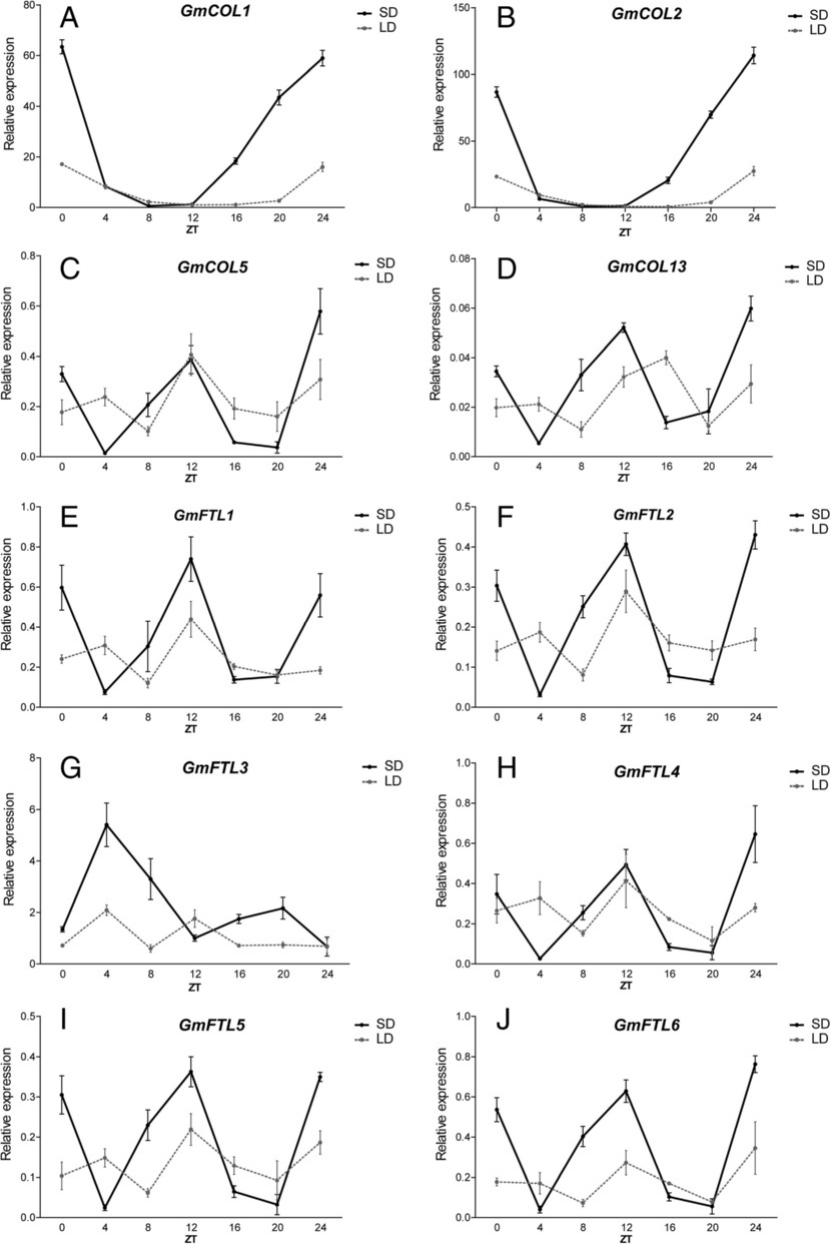
**The circadian rhythm expression of soybean *CO *and *FT *orthologs under SD or LD conditions. A** to **D**, the expression patterns of *GmCOL1, 2, 5, 13*, respectively. **E** to **J**, the expression patterns of *GmFTL1*-*6*, respectively. Seedlings were grown in SDs (8 h light/16 h dark cycles) or LDs (16 h light/8 h dark cycles) until the first trifoliolate leaf was fully expanded. Five trifoliolate leaves as one sample were collected at the times shown after dawn (ZT 0). Relative expressions were normalized to *GmUKNI* transcripts. Average and SD values for three replications are given for each data point.

To investigate the circadian rhythm of the candidate *CO-FT* regulon genes, transcriptions of 10 genes were detected in the leaves at the stage of the first trifoliolate fully opening under SD (8 h light/16 h dark) or LD (16 h light/8 h dark) conditions (Figure 5). Transcriptional circadian patterns of the paralog gene pair, *GmCOL1* and *2*, were very similar under both SD and LD conditions, and expression levels was much higher in SD conditions than LD conditions. Their expression peaks were present at dawn, and after that their expressions decreased until dusk (Figure 5A and B), which indicated that the two genes were strongly induced by darkness and inhibited by light. The expression rhythms of *GmCOL1* and *2* were similar to that of *Hd1* in rice, in which the abundance of *Hd1* mRNA was restricted to the dark period under SD conditions [65]. In addition, expression patterns of *LjCO*a, one of four *CO* homologs in *Lotus japonicus*, were also similar to that of *GmCOL1* and *2* under SD or LD conditions [66]. Compared to *GmCOL1* and *2*, *GmCOL5* and *13* were expressed at much lower level with different expression profiles in leaves (Figure 5C and D). In addition, the expression levels of *GmCOL5* were ten folds higher than those of *GmCOL13*, although they showed similar expression patterns under SD or LD conditions. Under SD conditions, expression peaks of *GmCOL5* and *13* occurred at dawn and ZT12, respectively. Under LD conditions, one expression peak of *GmCOL5* and *13* occurred at ZT4, and the other at ZT12 and ZT16, respectively (Figure 5C and D).

According to the circadian rhythm of six soybean *FT*-like genes (Figure 5E-J), five genes have similar expression patterns under SD conditions except *GmFTL3*. The expression of *GmFT1*/*2*/*4*/*5*/*6* occurred at dawn and peaked at ZT12 under SD conditions. But the expression peak of *GmFTL3* was at ZT4 (Figure 5G), which was consistent with previous reports in soybean [59,60] and in rice [8]. Under LD conditions, all six *GmFTL-like* genes showed similar expression rhythms (Figure 5E-J). For example, the expressions of six soybean *FT-like* genes reached to the maximum level at ZT4 and ZT12.

According to the diurnal rhythms of four soybean *CO* and six *FT* genes, *GmCOL5* and *13* showed similar expression patterns with *GmFTL1*, *2*, *4*, *5*, and *6*, indicating that the paralogous gene pair *GmCOL5* and *13* have important roles in regulation of expressions of *GmFTL1*, *2*, *4*, *5*, and *6*, and they may be composed of the soybean complicate and multiple *CO-FT* regulons to sense the circadian and photoperiodic signals.

### Ectopic activity on *Arabidopsis* flowering of *GmCOL*s and *GmFTL*s

**Figure 6:**
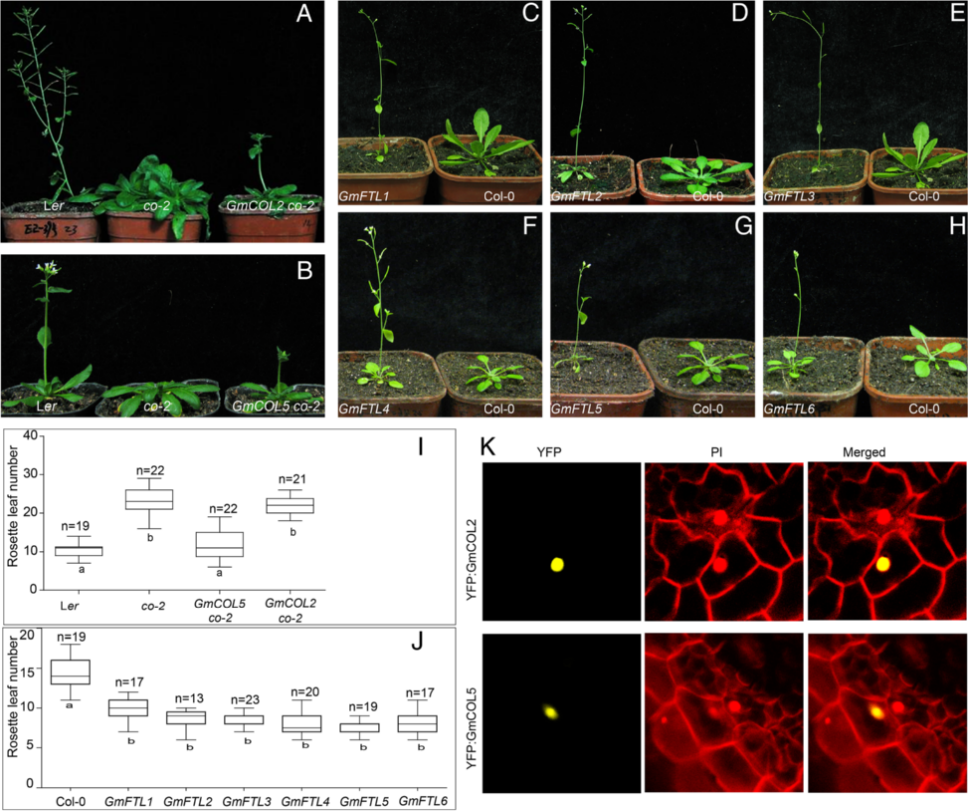
**Flowering function analysis in *Arabidopsis *and the subcellular localizations of two soybean *CO*-lineage genes. A** and **B** showed the phenotypes of overexpression of *GmCOL2* and *5* in *co-2* mutants, respectively. **C** to **H**, phenotypes of over-expression of *GmFTL1*, *2*, *3*, *4*, *5* and *6*, respectively. **I**, total rosette leaf numbers of transgenic lines of *GmCOL2* or *5* in *co-2* mutants. **J**, total rosette leaf numbers of transgenic lines of *GmFTL1*, *2*, *3*, *4*, *5* and *6* at flowering time. n, the total number of tested transgenic lines. Box plot showed total rosette leaf numbers of each line at the beginning of flowering and was generated using GraphPad Prism 5 software. The top of the box is the 75th percentile. The bottom of the box is the 25th percentile. The horizontal line intersecting the box is the median value of the group. Horizontal lines above and below the box represent maximum and minimum values, respectively. Boxes with dissimilar letters are significantly different at P < 0.01 after one-way analysis of variance (ANOVA). **K**, The subcellular localizations of GmCOL2 and 5 tagged by YFP at the N-terminal in the soybean leaves. PI (Propidium iodide) strain was selected to mark the cell walls.

In *Arabidopsis*, the *CO* paralog genes, *COL1* and *COL2*, have little effect on flowering time [67], and other members of BBX Cluster I genes, *COL3* and *COL5*, do not regulate the flowering time in *Arabidopsis*[68,69]. However, *COL9*, belonging to the BBX cluster II, is involved in regulation of flowering time by repressing the expression of *CO*, concomitantly reducing expressions of *FT* and delaying floral transition [70]. That indicates the functions of *CO-like* genes are not redundant in controlling the flowering time, and it may resulte from the rapid evolution of *CO-like* genes in plants [13]. To investigate the flowering functions of soybean *CO* orthologs, *GmCOL2* and *GmCOL5* under control of CaMV *35S* promoter were introduced into the *co* mutant (*co-2*), respectively. For *GmCOL2*, no significant changes in flowering time were detected in the over-expressing lines in LD conditions (Figure 6A and I). By contrast, over-expression of *GmCOL5* was able to rescue the late-flowering phenotype of *co* mutant (Figure 6B and I), indicating that *GmCOL5* gene may be a functional *CO* ortholog in soybean.

*FT* and its orthologs are the universal and conserved promoters of flowering in different plants [34,48,59,65,71]. Over-expressions of *GmFTL3* (*GmFTL2a*) or *4* (*GmFTL5a*) can promote the flowering in *Arabidopsis*[59,60]. To identify the flowering activity of soybean *FT-like* paralogs, all constructs of *GmFTL1* to *6* genes under control of CaMV *35S* promoter were respectively introduced into *Arabidopsis* ecotype Columbia (Col-0) (Figure 6C-H and I). Besides *GmFTL3* or *4*, other four soybean *FTL* genes can also change the flowering time of *Arabidopsis* (Figure 6C-H and I), suggesting that these paralogs of *FTL* genes may be functional *FT* orthologs in soybean. However, individual *GmFTL* genes had their own specific functions, because their spatio-temporal expression patterns were quite different.

In *Arabidopsis*, *TSF* and *FT* are differently regulated by distinct floral-inducing signals, so they show different functions on flowering in different conditions [56,61]. Functions of *GmTSF3*, *GmTSF4* and *GmPEBP21* in promoting flowering were further evaluated through heterologous over-expressions in *Arabidopsis* under LD conditions. The results showed that no significant changes in flowering time were detected in over-expression lines of *GmTSF3* and *GmTSF4*, compared to *Arabidopsis* wild type (Additional file 5), suggesting that they may not be the *FT-*lineage genes. Although *GmPEBP21* was much similar to *FT* in sequence (Additional file 4), it was not clustered into the *FT-like* (Figure 1B). And overexpression of *GmPEBP21* showed no effect on the flowering of *Arabidopsis* (Additional file 5), indicating that it also was not a functional *FT* gene.

### Conserved subcellular localization of soybean *CO* and *FT*-lineage proteins

**Figure 7:**
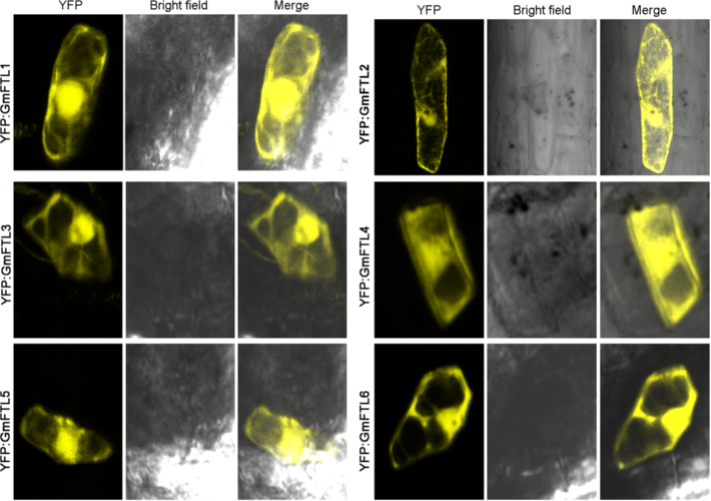
**Subcellular localizations of six soybean *FT *** **orthologs tagged by YFP at the N-terminal in the soybean leaves.**

Constructs of *GmCOL2*, *GmCOL5*, and *GmFTL1* to *6* genes tagged by a reporter gene (*YFP*) at the N- or C-terminal were employed to investigate the subsucellular localization through the particle bombardment in the young soybean leaves. Fluorescence signals of YFP-GmCOL2 and YFP-GmCOL5 were only present in the nucleus (Figure 6K), which were similar to CO homologous proteins in other species [14,20]. All six GmFTL proteins also resembled to *FT* homologous proteins in other plants [72,73] and localized in both the cytoplasm and the nucleus (Figure 7).

## Conclusion

BBX gene family contained seven clusters and the *CO*-homolog cluster were diverged from other clusters at the occurrence of plants. PEBP gene family had three groups and *FT*-lineage genes were diverged from *MFT*- and *TFL*-lineage genes at the occurrence of angiosperms. The role of the *CO-FT* regulon in photoperiodic regulation of flowering time was conserved, although the evolutionary rates of *CO*- and *FT*-lineage genes were different in angiosperms. In soybean, an ancient *CO*-lineage gene experienced three polyploidy events, and then formed four candidate of *CO* genes, *GmCOL1*, *2*, *5*, and *13*. Six *FT*-lineage genes, *GmFTL1*-*6*, were from an ancient locus prior to the WGT event. Based on the spatio-temporal expression profiles, *GmCOL1*/*2*/*5* and *GmFTL3*/*4*/6 were shown to play important roles in responses to photoperiod at the seedling stage. *GmCOL5*, *GmFTL1* to *6* showed flowering activity in *Arabidopsis*, suggesting that at least these genes may be the candidates of functional *CO-FT* regulons in soybean. Therefore, the *CO-FT* regulon in soybean was complicate and had multiple ones instead of a single one as in *Arabidopsis*, which may function synergistically in a spatio-temporal mode to control photoperiodic flowering.

## Methods

### Plant Materials

The soybean cultivar (Kennong18) was grown in the greenhouse under SD conditions (8 h light/16 h dark) at 24-28°C. The roots, hypocotyls, epicotyls, cotyledons, unifoliolate leaves and shoot apex (including the apical meristem and immature leaves) were sampled when the unifoliolate leaves were fully expanded (about two weeks after sowing). Other sample of the root, stem, unifoliolate leaves, various trifoliolate leaves, petiole and flower were harvested when the fourth trifoliolate were fully expanded (~45 days after sowing, flowering onset). Pods were sampled at 7, 14 and 21 days after flowering. For circadian samples, plants were grown in SD (8 h light/16 h dark) or LD (16 h light/8 h dark) conditions. When the first trifoliolate leaves were fully expanded, leaves were collected at 4 h intervals. All samples were immediately frozen in liquid nitrogen and stored at -80°C until use.

### Data sets and identification of the PEBP or BBX family

Protein sequences from the completely sequenced genomes were downloaded from Phytozome V8.0 (http://www.phytozome.net), including two monocots (*Oryza sativa* and *Z*ea *mays*), three eudicots (*Vitis vinifera*, *Arabidopsis thaliana*, and *Glycine max*), one lycophyte (*Selaginella moellendorffii*), one moss (*Physcomitrella patens*), and six chlorophytes (*Ostreococcus lucimarinus*, *Micromonas pusilla* RCC299, *M. pusilla* CCMP1545, *Coccomyxa subellipsoidea*, *Volvox carteri*, and *Chlamydomonas reinhardtii*). Additionally, sequences of four gymnosperms (*Picea sitchensis*, *Pinus radiate*, *Pinus pinaster*, and *Pinus sylvestris*) were gained from Protein Knowledgebase (http://www.uniprot.org/uniprot/).

In order to provide a uniform nomenclature for the B-box protein family, all the genes with B-box domain were classified as the BBX family [18]. HMMER 3.0 [74] was employed to identify the members of the BBX family (Pfam: PF00643) and the PEBP family (Pfam: PF01161) through their own profile-HMMs in 13 genomes.

### Phylogenetic analysis

Clustalw 2.0 (http://www.ebi.ac.uk/Tools/msa/clustalw2/) was used to aligned protein sequences of the BBX or PEBP family with default parameters. The reconstructions of phylogenetic trees were conducted through MEGA 5.0 [75]. Neighbour-joining (NJ) was used to construct different trees. To estimate evolutionary distances, the proportion of amino acids differences were computed using Jones-Taylor-Thornton (JTT) or Poisson correction models. To handle gaps and missing data, the pairwise-deletion option was selected. Bootstraps with 1000 replicates for Poisson correction model were performed to assess node support.

### Collinearity analysis of the soybean BBX or PEBP gene family

The modern soybean genome has experienced two “recent” whole-genome duplications (WGDs), and a more ancient triplication (Gamma WGT), and about 75% of the genes are present in multiple copies [41,76]. In soybean, the putative homologous chromosomal regions were identified by MCScanX [77] according to the alignment of protein sequences. For a protein sequence, the best five non-self hits in the soybean genome that met an E-value threshold of 10^-10^ were reported. And the homologous blocks including at least 5 collinear gene pairs and the gap number of gene pairs was not more than 20. The schematic diagrams for the collinearity of the members of BBX or PEBP family were drawn by Circos [78] (http://circos.ca/).

### Gene cloning and constructing expression vectors

The full CDS sequences of soybean *CO* orthologs (*GmCOL1*, *2*, *5*, and *13*), *FT* orthologs (*GmFTL1-6*), *GmTSF1-4*, and *GmPEBP21* were cloned into the entry vector (pGWCm) [79] and then recombined into appropriate destination vectors, pLEELA vector for overexpression in *Arabidopsis* or 2X35S∷Gateway cassette : YFP for the subcellular localization in soybean young leaves, with the Gateway technology (Invitrogen).

### Quantitative gene expression analysis

The procedure used for RNA extraction, cDNA synthesis, and PCR was as described by Hu, *et al*[80]. According to the specificity and efficiency of the primer pairs, the soybean *CO* or *FT* orthologs were designed by Beacon Designer 7.9, and at least one primer was specific for the target gene primer pairs (Additional file 6). Both *GmACT11* and *GmUKN1* were served as reference genes for the tissue-expression trials, and *GmACT11* was selected as the reference gene for the photoperiodic experiments.

### Transformation in *Arabidopsis* and growth conditions

Transformation of WT Col-0 and *co* mutant plants with *Agrobacterium bacteria* carrying recombinant constructs was performed using the floral dip method [81,82]. For each construct, at least three independent T1 lines were selected analyzed for flowering time under the LD condition (22-24°C, 150 μmol · m^-2^ sec^-1^).

### Subcellular localization

Transient expression of *GmCOL2*, *GmCOL5* and *GmFTL1* to *6* tagged by *YFP* in soybean young leaves was performed with a Model PDS-1000/He Biolistic Particle Delivery System (Bio-Rad). 10 micrograms of purified plasmids were coated with 500 μg 1 μm-gold particles, as described by the manufacturer. After bombardment, young soybean leaves were incubated overnight at 25°C on solid 1/2 MS medium. Fluorescent cells were imaged by confocal microscopy (Leica TCS SP5, Leica Microsystem, Wetzlar, Germany). YFP was excited by the 514-nm argon laser line, and PI (Propidium iodide) stain was excited using a 561-nm He-Ne laser. Fluorescence was detected using photomultiplier tube settings as follows: YFP (520 to 560 nm), and PI (570 to 620 nm). At last, post-acquisition image analyzing and processing were performed using MBF ImageJ (version 1.46) (https://www.macbiophotonics.ca/).

## Authors’ contributions

CF carried out all the analysis and interpreted the results, and wrote the manuscript. RH, XZ, CF carried out experiments of *GmFTL*s. XW gave some good advices on writing the manuscript. WZ, QZ, JM and CF done some works of *GmCOL*s. YF conceived the project, supervised the analysis and critically revised the manuscript. All authors read and approved the final manuscript.

## Supplementary Material

Additional file 1 **The information of the BBX or PEBP family.** Sheet Gm, At, Vv, Os, Zm, Pp, and Sm showed the information of *G. max*, *A. thaliana*, *V. vinifera*, *O. sativa*, *Z. mays*, *P. patens* and *S. moellendorffii*, respectively; Sheet Gymnosperm included *P. sitchensis*, *P. radiate*, *P. pinaster*, and *P. sylvestris*; Sheet Algae included *O. lucimarinus*, *M. pusilla* RCC299, *M. pusilla* CCMP1545, *C. subellipsoidea*, *V. carteri*, and *C.reinhardtii*. Click here for file

Additional file 2 The best match sequences of motifs for the BBX or PEBP family. Click here for file

Additional file 3 **Spatio-temporal expressions of *GmFTL7.*** R, root; H, hypocotyl; C, cotyledon; E, epicotyl; U, unifoliolate leaf; S, stem; T1, T2, T3, T4, the first, second, third, and fourth trifoliolate leaf, respectively; F, flower; SAM, the shoot apex (including the apical meristem and immature leaves) at the seedling stage. P1, P2, and P3: seven, fourteen and twenty one days after the onset of flowering, respectively. The geometric means of *GmACT11* and *GmUKNI* transcripts were used as the reference transcript. The bars are means of three replicates, and each replicate represented a pool from at least five plants, and means was formulated as ⊿Ct = Ct_(Target gene)_-Ct_(geometric means of reference genes)_. Click here for file

Additional file 4 **The similarity between soybean and Arabidopsis *FT-like *** **genes.** Click here for file

Additional file 5 **Phenotype of *GmTSF3*, *GmTSF4 *** **and *GmPEBP21 *** **over-expressing in *Arabidopsis. *** **A**, The phenotype of transgenic lines. **B**, The rosette leaf number of the transgenic lines at flowering. n showed the total detected lines. Box plot showed total rosette leaf numbers of each line at the beginning of flowering and was generated using GraphPad Prism 5 software. The top of the box is the 75th percentile. The bottom of the box is the 25th percentile. The horizontal line intersecting the box is the median value of the group. Horizontal lines above and below the box represent maximum and minimum values, respectively. Click here for file

Additional file 6 **The primers of soybean *CO *** **or *FT*-lineage genes.** Click here for file
