# Supplementary material for: Conserved CO-FT regulons contribute to the photoperiod flowering control in soybean
Source: BMC Plant Biol. 2014 Jan 7;14:9. doi: 10.1186/1471-2229-14-9 (PMC3890618; doi:10.1186/1471-2229-14-9)
Supplement: Additional file 3 — Spatio-temporal expressions of GmFTL7. R, root; H, hypocotyl; C, cotyledon; E, epicotyl; U, unifoliolate leaf; S, stem; T1, T2, T3, T4, the first, second, third, and fourth trifoliolate leaf, respectively; F, flower; SAM, the shoot apex (including the apical meristem and immature leaves) at the seedling stage. P1, P2, and P3: seven, fourteen and twenty one days after the onset of flowering, respectively. The geometric means of GmACT11 and GmUKNI transcripts were used as the reference transcript. The bars are means of three replicates, and each replicate represented a pool from at least five plants, and means was formulated as ⊿Ct = Ct(Target gene)-Ct(geometric means of reference genes). [file 1471-2229-14-9-S3.docx]

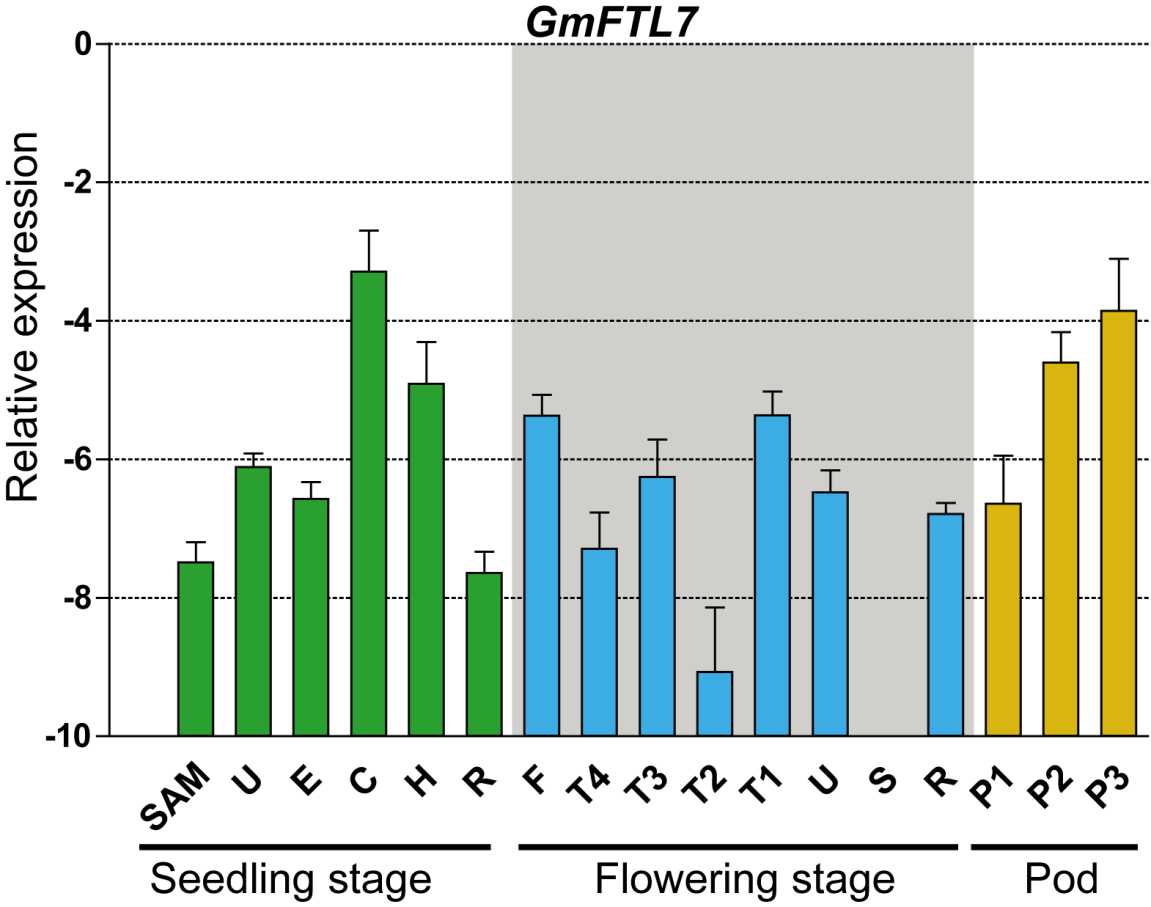


**Additional file 3.** Spatio-temporal expressions of *GmFTL7*. R, H, C, E, U, S, T1, T2, T3, T4, F, SAM: the root, the hypocotyl, the cotyledon, the epicotyl, the unifoliolate leaf, the stem, the first trifoliolate leaf, the second trifoliolate leaf, the third trifoliolate leaf, the fourth trifoliolate leaf and the flower, the shoot apex (including the apical meristem and immature leaves) at the seedling stage, respectively. And P1, P2, and P3: seven, fourteen and twenty one days after the onset of flowering, respectively. The geometric means of *GmACT11* and *GmUNKI* transcripts were used as the reference transcript. The bars are means of three replicates, and each replicate represented a pool from at least five plants, and means was formulated as ⊿Ct= Ct_(Target gene)_-Ct_(geometric means of reference genes)_.
