# Supplementary material for: Conserved CO-FT regulons contribute to the photoperiod flowering control in soybean
Source: BMC Plant Biol. 2014 Jan 7;14:9. doi: 10.1186/1471-2229-14-9 (PMC3890618; doi:10.1186/1471-2229-14-9)
Supplement: Additional file 4 — The similarity between soybean and Arabidopsis FT-like genes. [file 1471-2229-14-9-S4.docx]

Additional file 4 The similarity between soybean and Arabidopsis *FT-like* genes

| Gene symbol | Locus name | Protein  Size (aa) | *Arabidopsis*  homolog locus^b^ | *Arabidopsis*  locus description | Identity^b^ | E-value^b^ |
| --- | --- | --- | --- | --- | --- | --- |
| *GmFTL1* | *Glyma16g04840* | 175 | *AT1G65480* | *FT* | 67% | 4E-67 |
| *GmFTL2* | *Glyma19g28390* | 175 | *AT1G65480* | *FT* | 73% | 1E-74 |
| *GmFTL3* | *Glyma16g26660* | 176 | *AT1G65480* | *FT* | 68% | 9e-61 |
| *GmFTL4* | *Glyma16g04830* | 172 | *AT1G65480* | *FT* | 66% | 1E-65 |
| *GmFTL5* | *Glyma16g26690* | 176 | *AT1G65480* | *FT* | 68% | 2E-67 |
| *GmFTL6* | *Glyma19g28400* | 172 | *AT1G65480* | *FT* | 66% | 3E-65 |
| *GmTSF1* | *Glyma18g53690* | 173 | *AT4G20370* | *TSF* | 69% | 3E-68 |
| *GmTSF2* | *Glyma18g53680* | 176 | *AT4G20370* | *TSF* | 68% | 1E-64 |
| *GmTSF3* | *Glyma08g47820* | 177 | *AT1G65480* | *FT* | 66% | 5E-66 |
| *GmTSF4* | *Glyma08g47810* | 171 | *AT1G65480* | *FT* | 64% | 3E-65 |
| *GmPEBP21* | *Glyma08g28470* | 171 | *AT1G65480* | *FT* | 51% | 2E-31 |

^­^Note: ^a^Gene locus information is from Phytozome (<http://www.phytozome.net/cgi-bin/gbrowse/soybean/>); ^b^Local Balstp was carried out against Arabidopsis protein database (TAIR 10); *GmTSF3* and 4 were in red, showing showed much similar sequences with *FT* than that of *TSF*, and collinear with *GmTSF1* and 2.
