# Supplementary material for: Conserved CO-FT regulons contribute to the photoperiod flowering control in soybean
Source: BMC Plant Biol. 2014 Jan 7;14:9. doi: 10.1186/1471-2229-14-9 (PMC3890618; doi:10.1186/1471-2229-14-9)
Supplement: Additional file 5 — Phenotype of GmTSF3 , GmTSF4 and GmPEBP21 over-expressing in Arabidopsis. A, The phenotype of transgenic lines. B, The rosette leaf number of the transgenic lines at flowering. n showed the total detected lines. Box plot showed total rosette leaf numbers of each line at the beginning of flowering and was generated using GraphPad Prism 5 software. The top of the box is the 75th percentile. The bottom of the box is the 25th percentile. The horizontal line intersecting the box is the median value of the group. Horizontal lines above and below the box represent maximum and minimum values, respectively. [file 1471-2229-14-9-S5.docx]

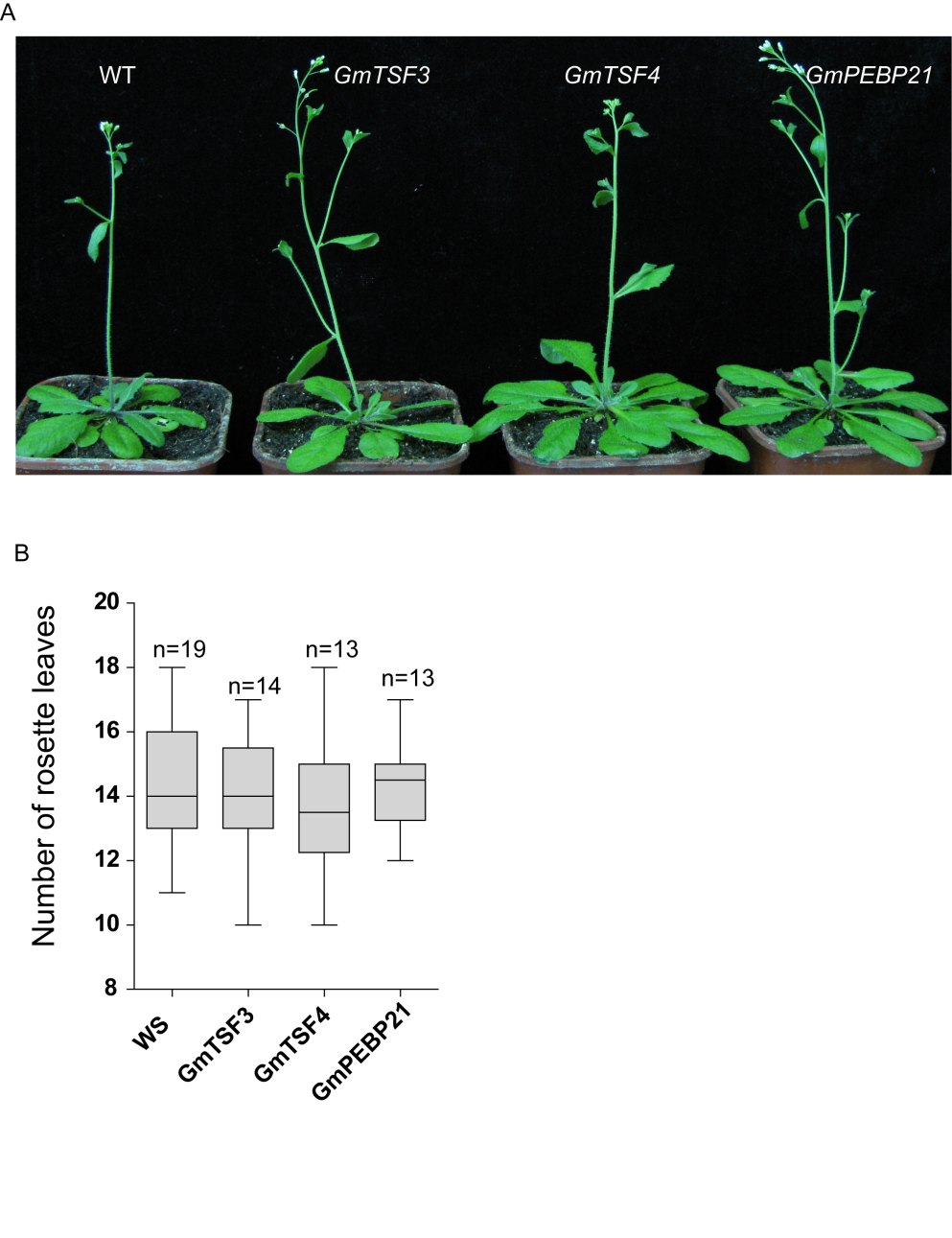


**Additional file 5.** Phenotype of *GmTSF3*, *GmTSF4* and *GmPEBP21* over-expressing in *Arabidopsis*. A, The phenotype of transgenic lines. B, The rosette leaf number of the transgenic lines at flowering. n showed the total detected lines. Box plot showed total rosette leaf numbers of each line at the beginning of flowering and was generated using GraphPad Prism 5 software. The top of the box is the 75th percentile. The bottom of the box is the 25th percentile. The horizontal line intersecting the box is the median value of the group. Horizontal lines above and below the box represent maximum and minimum values.
